# Quality of life and challenges experienced by the surviving adults with transfusion dependent thalassaemia in Malaysia: a cross sectional study

**DOI:** 10.1186/s12955-021-01897-4

**Published:** 2022-01-08

**Authors:** Wai Cheng Foong, Kooi Yau Chean, Fairuz Fadzilah Rahim, Ai Sim Goh, Seoh Leng Yeoh, Angeline Aing Chiee Yeoh

**Affiliations:** 1 Department of Paediatrics, RCSI & UCD Malaysia Campus (formerly Penang Medical College), 4, Jalan Sepoy Lines, 10450 George Town, Penang Malaysia; 2 Department of Family Medicine, RCSI & UCD Malaysia Campus (formerly Penang Medical College), 4, Jalan Sepoy Lines, 10450 George Town, Penang Malaysia; 3 Department of Public Health, RCSI & UCD Malaysia Campus (formerly Penang Medical College), 4, Jalan Sepoy Lines, 10450 George Town, Penang Malaysia; 4 Haematology Unit, Hospital Pulau Pinang, Ministry of Health Malaysia, Jalan Residensi, 10990 George Town, Penang Malaysia; 5 Department of Paediatrics, Hospital Pulau Pinang, Ministry of Health Malaysia, Jalan Residensi, 10990 George Town, Penang Malaysia; 6 Department of Paediatrics, Hospital Seberang Jaya, Ministry of Health Malaysia, Jalan Tun Hussein Onn, 13700 Seberang Jaya, Penang Malaysia

**Keywords:** Thalassaemia, Quality of life, Healthy days, Employment

## Abstract

**Background:**

Improvement in medical management has enabled transfusion dependent thalassaemia (TDT) patients to survive beyond childhood, building families, and contributing to the labour force and society. Knowledge about their adult life would provide guidance on how to support their needs. This study aims to explore the general well-being of adults with TDT, their employment status and challenges.

**Methods:**

This study recruited 450 people with TDT, aged 18 and above, of both genders through all regional Thalassaemia societies in Malaysia and from the two participating hospitals, over five months in year 2016. A self-administered questionnaire including ‘Healthy Days Core Module’, WHOQOL-BREF and employment measurements was used. Multiple linear regression models were fitted with associations adjusted for several potential confounders.

**Results:**

A total of 196 adults with TDT responded to the survey (43.6% response rate). Almost half (45%) had comorbidities and 9% suffered multiple complications: bone-related (13%), hormonal (12%), cardiac (3%) and infections (2%), resulting in 23% seeking treatment more than twice monthly. Within a month, they suffered from at least three days with poor physical and or mental health and their normal daily activities were disrupted up to three days. 36% were jobless and 38% of those with a job were receiving salaries below RM1000. The mean WHOQOL-BREF score (mean (SD)) was: physical health 62.6 (15.5), psychological health 64.7 (15.7), social relationship 64 (15.9), environmental health 60.8 (16.7). Having days with mental issues, financial status, education level, ethnic and marital status were main factors affecting QOL scores. Open questions showed dissatisfaction with health service provision, conflicting judgement in prioritising between health and job, and poor public empathy.

**Conclusion:**

The adults with TDT perceived their health as good and had less unhealthy days when compared with people with other chronic diseases. However, some perceived themselves to be facing more life disruption in a rather non-supportive community and that health services do not meet their needs. Future qualitative studies are needed to focus on their perceived needs and to look for more tailored supportive approaches.

## Plain English Summary

**What is the problem?:**

With advancements in medical care, more people with transfusion dependent thalassaemia (TDT) reached adulthood, something which was uncommon decades ago. They should have similar physical-mental capabilities as people without TDT. However, the stigma of their condition has remained and their potential capabilities have often been underestimated by the public. We need to understand their adult life in order to assist them further.

**What did we do?:**

We surveyed 196 TDT patients, using a set of questionnaires. We enquired of their perceptions about their health status and quality of life (QOL: physical, mental, social relationship, environment). We asked them open questions on challenges they were facing during adulthood and employment.

**What did we find?:**

Some participants (45%) were found to have other medical problems and that they could be feeling unwell at least three days in a month. Participants scored above 60 out of 100 for all of the QOL aspects (a higher score denotes a better QOL). We noted some continued to lose career advancement opportunities, face discrimination and had difficulties managing their health, in a rather non-supportive community.

**What is our conclusion?:**

People with TDTs were doing relatively well as young adults. Life challenges persist. More qualitative studies are needed to understand their challenges better and to look for more tailored supportive approaches.

## Introduction

Thalassaemia is the commonest inherited blood disorder in Malaysia with a total population of 8681 in 2018. This condition is characterised by low haemoglobin levels, due to defects in either the alpha- or beta-globin chain of the red blood cells, leading to an imbalance in globin chain, thus destruction of the red blood cell and ultimately, low haemoglobin presenting as anaemia [1]. The commonest clinical subtype of thalassaemia in Malaysia is Hb E-beta thalassaemia (34.37%), followed by beta thalassaemia major (33.52%), Hb H disease (18.26%), beta thalassaemia intermedia (9.37%) and others (4.48%) [2]. People with a more severe spectrum will require 3– 4-weekly blood transfusions for survival and are categorized as havingtransfusion dependent thalassaemia (TDT).

Beta thalassaemia major followed by HbE-beta thalassaemia have the most serious clinical presentation with poor growth, chronic lethargy and organ failure from chronic anaemia [3]—taking its toll on a person's physical capabilities when medical management is inadequately provided. Life-long regular blood transfusion is required for survival from a young age, when they have to endure hours of blood transfusion and later in life, when iron overload occurs due to transfusion, hours of chelation with Deferoxamine, the infusion type of iron chelating agent. Otherwise, they would have to ingest oral iron chelating agents which could be unpalatable or unaffordable to some. The required medical attention causes their daily activities to be disrupted with a life bound by activities that required their presence at the hospital. This disruption in life is also costly. They have to be responsible for themselves in managing their own health, finances and family when they reach adulthood—which had been a huge burden experienced by their caretakers when they were young [4]. Without optimal treatment and responsibility, not only will their growth be affected in terms of distorted body images such as short stature, thalassaemic facies and 'child-like' appearance [4], they could also face social stigmatization from 'frequent absenteeism' or poor physical performance, which then lowers their self-esteem and causes mental stress [5].

The prolongation of life expectancy for people with TDT through advancement and availability of necessary treatment has enabled them to experience adulthood [6–8]. In 2018, 92% of thalassaemics registered in the National Registry were still alive in Malaysia and around 44% have reached adulthood. They have been receiving assistance such as provision of free medical treatment and mass community screening [9, 10]. The TDT population continues to grow. Although there is a decline in the number of new cases, many more will be entering adulthood soon [9].

A report has shown that the global disability-adjusted life years increased by 40% for non-communicable diseases when life-expectancy is prolonged [11]. This could be a warning of the future that people surviving with TDT will have to face. People with TDTs are known to have expressed their concerns about how they have struggled with life and how they have been marginalized [5, 12], but there were limited studies, if any, about their actual adulthood needs, employment and perception of healthcare services. The perception of having thalassaemia has also been negative since childhood, a burden to the patient, family and nation [4, 13]. The social media continue to publish their challenges intermittently despite the government’s contemporary assistance [14–17]. Therefore, it is timely to explore how they fare during adulthood in the era when much medical assistance is provided. We conducted this study to determine their clinical and psychosocial burden, including employment status and their actual needs during adulthood. We also aimed to assess if there is another area of focus for the healthcare team to support TDTs during their transition to adulthood.

## Methods

### Study design

This was a cross sectional study and eligible participants were recruited through convenient sampling.

Patients from the thalassaemia units of two hospitals in Penang, Malaysia and or members from all registered regional thalassaemia societies in Malaysia were approached face-to-face by their attending doctor, nurse or selected regional site coordinators who worked for the regional thalassaemia society from all over Malaysia between August 2016 and December 2016.

Those in-charge of recruitment (research assistants, RA) were briefed about the aims and conduct of the study, and trained on recruitment process. Once a written consent was obtained, participants were given an option to complete the questionnaire either by an electronic link to Google form or a hard printed copy. The questionnaires were to be self-completed without any assistance or interference from RA. Participants were given up to an hour to complete the questionnaire. The completed questionnaires in hard copies were then sealed by the participant in an envelope, which would then be collected, compiled and send as bulk mail back to the investigators by the RA. As for the electronic questionnaires, all submissions were done online and the responses were automatically stored at a pre-set google form which could only be accessed by investigators.

### Sample size estimation

We used a survey sample size calculator according to sample size formula for a finite population. A sample size of 344 respondents was determined from a level of confidence set at 95%, a margin of error at 5%, a population proportion of 0.5, and a population size of 3200 (from the estimated adult TDT registered in Malaysia [18, 19]. A total of 450 questionnaires were distributed to account for the possibility of incomplete information.

### Participants (inclusion and exclusion criteria)

Participants were recruited when they fulfilled the inclusion criteria of having transfusion dependent thalassaemia (TDT) as diagnosed by their doctors at the time of recruitment, and aged 18 years and above. Those who were not capable of answering the questionnaire independently were excluded.

### Assessment and outcome measures

The structured questionnaire consisted of four sections on (a) socio-demographics characteristics, (b) their health status and well-being, (c) quality of life and (d) free text on employment, challenges and healthcare systems.

Details of the self-administered questionnaire are as follows:

- Socio-demographic characteristics: Age, gender, race, type of thalassaemia, co-morbidity, medication, marital status, education, income and occupation.
- Healthy Days Core Module (CDC HRQOL-4) [20, 21]: This is part of the National Health and Nutrition Examination Survey (NHANES) used in the National Commission for Quality Assurance's (NCQA) Healthcare Effectiveness Data and Information Set (HEDIS), and in this study, the focus was on thalassaemia. There are 4 self-rated questions. The first question is on respondent's perception of own general health in a 5-point Likert scale and the subsequent questions require the respondents to estimate the number of days they felt unhealthy physically, mentally and when daily activities were limited, for the past 30 days.
  This set of questionnaires is only available in English and was translated to Malay and Chinese languages. The translations were cross-checked and back translated to ensure accurate and true representation of the original document.
- WHOQOL-BREF [22]: This is an abbreviated version of the World Health Organization Quality of Life questionnaire (WHOQOL 100) which comprised of two individual questions on perception and satisfaction in QOL, and questions that assessed four QOL domains (physical health, psychological health, social-relationship, environmental health). Responses are graded on a scale 1–5. Each response for a particular domain was then added up to form a cumulative score that has a maximum value of 100. A higher score indicates a better QOL for a particular domain. This set of questionnaires is available in three languages: English, Malay and Chinese, which were used.
- Perception of healthcare services and employment: The questions included job descriptions, job’s satisfaction, monthly salary, monthly off-days, support at work, discrimination at work, disclosure of disease and willingness to disclose disease with colleagues. Participants were given the opportunity to elaborate on any questions asked including areas which could be related to healthcare services in the form of free field texts.

Both the questions on biodata and perception of healthcare services and employment were developed by the investigators using an iterative process, piloted on a small sample of adult thalassaemia patients and modified according to feedback obtained. The questionnaire was made available in the three major languages used in Malaysia: English, Malay and Chinese.

Participants were given a choice to choose depending on their language literacy.

### Endpoints

The study endpoints focused on the participants perception of general health and quality of life, and their perception on healthcare services and employment. This study then further identified factors that were significantly associated with the quality of life of these TDT participants.

### Statistical analysis

In reporting descriptive statistics, continuous data was presented as mean with standard deviation (SD) and categorical data presented as frequency (n) with percentage (%). Chi-square analysis (X^2^) was conducted to compare the baseline characteristics according to the age at diagnosis and the age receiving first blood transfusion. Data obtained from Healthy Days Core Module and WHOQOL-BREF were displayed using the respective intended unit or scores.

Linear regression analysis was used to determine potential factors associated with their QOL. All study factors were identified as clinically important variables. Simple linear regression was used to identify possible factors associated with QOL. The factors were then fitted into multiple linear regression models based on each QOL domain, adjusting to the effect of other study factors. The results from linear analysis were reported as coefficient, its 95% confidence interval (CI) and *p*-value. All statistical analysis was done using Stata-13 [23]. A *p*-value of less than 0.05 was taken as a statistically significant finding.

## Results

Of the 450 questionnaires distributed to 13 states of Malaysia, 217 were returned which accounted for 43.6% response rate. Twenty-one responses were excluded because of less than 80% completion of the questionnaire. The final number of questionnaires analysed was 196. The responders came from nine out of the 13 states.

There were fairly equal proportion of males (44%) and females (56%) participants. The mean age was 27 (SD 7.4). They were mainly of the Malay ethnicity (62%). The majority of them suffered from beta thalassaemia major (64%), followed by HbE beta thalassaemia (30%) and HbH disease) (6%) (Table 1).

**Table 1:** Socio-demographic characteristics (n = 196)

Characteristics	n (%)
Age in years^a^	27 (7.4)
Gender	
Male	87 (44)
Female	109 (56)
Ethnic	
Malay	121 (62)
Sabah and Sarawak indigenous (e.g. Kadazan-Dusun)	40 (20)
Chinese	35 (18)
Types of thalassaemia	
Beta thalassaemia major	126 (64)
HbE beta thalassaemia	59 (30)
HbH disease	11 (6)
Types of comorbidity (n = 179)	
Musculoskeletal	23 (13)
Endocrine	21 (12)
Cardiovascular	6 (3)
Blood infection	4 (2)
Psychological	4 (2)
Multiple	16 (9)
Others	6 (3)
Medication	
Iron chelators	191 (97)
Hormonal medication	110 (56)
Cardiac medication	8 (4)
More than 1 group of medication	157 (80)
Mode of administration of iron chelating agents	
Subcutaneous only	25 (13)
Oral only	78 (40)
Combined oral and subcutaneous	88 (45)
None needed	5 (3)
Frequency of hospital visits (n = 194)	
Once a month	80 (41)
Twice a month	70 (36)
More than twice a month	44 (23)
Monthly income (n = 172, included those participants with a previous employment)	
Less than RM1000	62 (36)
Between RM1000 and RM2000	60 (35)
More than RM2000	21 (12)
Unemployed	24 (14)

^a^Mean (SD)

We found that 45% of the participants were suffering from at least one complication of thalassaemia or the treatment they had received (Table 1). Highest co-morbidity on the list was musculoskeletal problems (13%) such as osteoporosis-osteopenia syndrome, bone pain or fractures or disfigurements, followed by endocrine issues (12%) such as diabetes mellitus, hypothyroidism or infertility and cardiac abnormalities (3%). Other health issues included transfusion related infections (2%) and psychological problems (2%) such as depression and anxiety. Among the participants, 8% had various types of complications and another 9% did not make any disclosure. Hence, 82% of participants were on some form of medication in addition to their regular blood transfusion and folate supplements. Almost all (97%) participants were on iron chelating agents to prevent iron overload (Table 1).

### Effects of having chronic anaemia and dependency on blood transfusion

We found 62% of those participants with beta thalassaemia major to be diagnosed and transfused before the age of 2 years. Another 12% were diagnosed as having thalassaemia before the age of 2 years but started their first transfusion after 2 years old and the remaining 26% were diagnosed and had first transfusion after 2 years (Table 2). The majority of those with thalassaemia intermedia (HbE beta thalassaemia and HbH disease) manifested much later and 3.5% needed medical care before age of 2 years. At the time of this study, all participants were transfused at least once a month and these transfusions were conducted at hospitals only on weekdays during office hours. Further hospital visits were expected for various reasons such as doctor/pharmacist consultations and investigation procedures, thus causing 58% of the participants to require hospital visits twice or more per month. There were significantly more singles among those who were diagnosed and were transfused blood earlier (p ≤ 0.001) but no significant association between the timing of diagnosis and transfusion with co-morbidity, education and employment status. (Table 2).

**Table 2:** Chronic anaemia and dependency on blood transfusion (n = 196)

Characteristics	Total (n = 196)	*p* value	Time of diagnosis and first transfusion		
			^a^ Group 1 (n = 85)	^b^ Group 2 (n = 22)	^c^ Group 3 (n = 89)
Type of thalassaemia		0.000			
Beta thalassaemia major	126		78	15	33
HbE beta thalassaemia	59		7	7	45
HbH disease	11		0	0	11
Presence of other comorbidities		0.135			
Yes	77		41	8	28
No	102		39	14	49
Marital status		0.001			
Married	41		8	4	29
Single	154		77	18	59
Education		0.133			
Primary	13		7	2	4
Secondary	133		60	10	63
Tertiary	50		18	10	22
Employment status		0.147			
Employed	126		57	10	59
Unemployed	70		28	12	30

^a^Group 1: Thalassaemia diagnosed and started transfusion before age of 2 years

^b^Group 2: Thalassaemia diagnosed before 2 years old but first transfusion after 2 years old

^c^Group 3: Thalassaemia diagnosed and started transfusion after 2 years old

### Perception of general health and quality of life

In general, the participants considered their health to be between 'Good' and ''Very Good'. Within 30 days, the participants experienced an average (mean (SD)) of 5.6 (5.2) unhealthy days, whereby a mean (SD) of 3.1 (3.0) days could be due to being physically unwell and 2.6 (3.5) days being mentally unwell. We found a mean (SD) of 2.5 (3.3) days when their daily activities were affected and limited.

The overall perception of QOL rating and health satisfaction was 3.7 (0.8) and 3.5 (0.7) on a Likert scale of 1–5 with the value 5 as excellent or very satisfied. For perception in QOL ratings, majority of the participants rated themselves to be in a very good QOL state (53%), followed by good (33%), excellent (11%), very poor (2%) and poor (1%). Most of them were satisfied with their health (47%), another 41% were neither satisfied nor dissatisfied, the remaining rated very satisfied (7%), dissatisfied (4%) and very dissatisfied (1%). Their quality of life across all 4 domains was above 60 out of a maximum score of 100, with psychological health obtaining the highest score at 64.7 (15.7) and environmental health getting the lowest score at 60.8 (16.7) (Table 3).

**Table 3:** Perception of healthy days and quality of life (QOL)

	Mean (SD)
Healthy Days Core Module (CDC HRQOL-4)	
"In general, would you say your health is…"^#^	3.01 (0.79)
Number of days affected by physical issues (over 30 days) (n = 190)	3.1 (3.0)
Number of days affected by mental issues (over 30 days) (n = 189)	2.6 (3.5)
Number of days when activities were limited by physical and or mental issues (over 30 days) (n = 191)	2.5 (3.3)
Summary index of unhealthy days (overall unhealthy days) (over 30 days) (n = 196)	5.6 (5.2)
WHOQOL-BREF	
Overall perception on QOL rating* "How would you rate your quality of life?"	3.7 (0.8)
Overall health satisfaction rating** "How satisfied are you with your health?"	3.5 (0.7)
Domain 1: Physical health*** (n = 189)	62.6 (15.5)
Domain 2: Psychological health*** (n = 189)	64. 7 (15.7)
Domain 3: Social relationship*** (n = 188)	64.0 (15.9)
Domain 4: Environmental health*** (n = 186)	60.8 (16.7)

^#^Likert scale: 5 = Excellent, 4 = Very good, 3 = Good, 2 = Fair, 1 = Poor

*Likert scale: 5 = Excellent, 4 = Very good, 3 = Neither poor or good, 2 = Poor, 1 = Very poor

**Likert scale: 5 = Very satisfied, 4 = Satisfied, 3 = Neither dissatisfied or satisfied, 2 = Dissatisfied, 1 = Very dissatisfied

***A score closer to 100 indicates a better quality

### Perception on healthcare services and employment

At the time of the study, 64% participants held a job. Majority (44%) earned between RM1000 and RM2000 a month, followed by 38% earning below RM1000 a month and 3% participants did not disclose their monthly earnings. 6% of the working participants were not confident in retaining their current employment. The remaining (36%) participants were not earning a living because they had never held any job (27%), had been retrenched for the past 2 years (39%) or were students (34%). Of those participants with working experiences, 41% were willing to disclose their thalassaemia status, 82% had shared their thalassaemia condition with colleagues, 34% had received satisfactory workplace support, 77% had experienced difficulties in their job because of thalassaemia and 57% reported discrimination for having thalassaemia. There was no significant association between their perception of employment difficulties because of thalassaemia and the number of overall unhealthy days (*p* = 0.32), or between the number of days when activities were affected by physical and or mental issues and their perception of employment difficulties because of thalassaemia (*p* = 0.06). Among those with working experience, at least 12% have chosen not to give a response to at least one of the questions in this section.

There were 67 responses to the free text comments on their healthcare services and employment. The main response was a negative perception of having thalassaemia and how it had affected their job potentials (35 responses), followed by 21 responses which pleaded for more employment opportunities or promotions for people with thalassaemia and less workforce discrimination. The reasons for the discrimination were their frequent absenteeism from work, easy fatigue and being short or ugly. There were six responses expressing dissatisfaction with health service provision such as non-flexi treatment hours or no dedicated clinics, 20 responses on conflicting judgement or ability in prioritising between health and job, and 18 responses about poor public empathy were expressed. Six participants wanted more community awareness and support together with a better health professional-patient relationship in order to enable them to cope with having thalassaemia and another five participants wanted assistance in planning for their future.

### Factors associated with quality of life (WHOQOL-BREF)

Table 4 showed the factors associated with QOL. Results from the simple linear regression showed that there were three main factors significantly associated with QOL: having days with mental issues (reduced the scores for all four domains), higher education (increased the physical health, social relationship and environmental health scores) and higher monthly income (increased the physical health and social relationship scores) (Table 4).

**Table 4:** Linear regressions on sociodemographic, CDC healthy-days and employment associated with WHOQOL-BREF scores

Factors	Simple linear regression (coefficient (95% CI))	Multiple linear regression^a^ (coefficient (95% CI))
Physical health		
Education (higher)	5.17 (1.01, 9.34)*	2.14 (− 2.35, 6.64)
Hospital visits (more frequent)	− 3.78 (− 6.54, − 1.02)*	− 0.70 (− 3.61, 2.20)
Monthly income (higher)	3.60 (1.05, 6.15)*	4.38 (1.11, 7.66)*
Days when activities were affected by physical and or mental issues (more days)	− 1.58 (− 2.26, − 0.90)*	− 1.98 (− 2.92, − 1.05)*
Days affected by physical issues (more days)	− 1.13 (− 1.92, − 0.33)*	− 0.39 (− 1.42, 0.64)
Days affected by mental issues (more days)	− 1.02, (− 1.7, − 0.35)*	− 0.25 (− 1.02, 0.53)
Psychological health		
Malay ethnic	4.01 (1.15, 6.87)*	4.54 (0.83, 8.24)*
Presence of other comorbidities	− 5.40 (− 9.98, − 0.82)*	− 5.43 (− 10.88, 0.02)
Days when activities were affected by physical and or mental issues (more days)	− 1.03 (− 1.75, − 0.32)*	− 1.50 (− 2.55, − 0.44)*
Days affected by mental issues (more days)	− 1.04 (− 1.72, − 0.35)*	− 0.50 (− 1.37, 0.37)
Social relationship		
Education (higher)	5.26 (0.92, 9.61)*	3.51 (− 1.36, 8.37)
Marital status (being single)	− 7.58 (− 13.15, − 2.00)*	− 12.27 (− 18.75, − 5.77)*
Monthly income (higher)	3.97 (1.38, 6.56)*	3.64 (0.14, 7.14)*
Days affected by mental issues (more days)	− 1.10 (− 1.78, − 0.43)*	− 0.86 (− 1.68, − 0.05)*
Environmental health		
Education (higher)	7.01 (2.49, 11.53)*	4.90 (0.01, 9.90)
Malay ethnic	3.23 (0.11, 6.36)*	4.54 (0.89, 8.18)*
Hospital visits (more frequent)	− 3.57 (− 6.62, − 0.53)*	− 0.70 (− 3.91, 2.50)
Presence of other comorbidities	− 6.34 (− 11.23, − 1.44)*	− 5.11 (− 10.45, 0.22)
Days affected by mental issues (more days)	− 0.97 (− 1.72, − 0.23)*	− 0.48 (− 1.34, 0.37)
Currently have a job	− 4.72 (− 9.74, 0.31)*	− 2.68 (− 9.08, 3.72)
Monthly income (higher)	1.36 (− 1.47, 4.20)	3.75 (0.14, 7.37)*

Factors which have no significant association in both simple and multiple linear regression were not displayed for the specified domain

*p < 0.05

^a^Adjusted according to gender, ethnicity, education, marital status, age at first diagnosis and transfusion, type of iron chelation, frequency of hospital visits, presence of other comorbidities, employment status (monthly income, currently have a job) and factors in CDC healthy-days (days affected by physical issues, days affected by mental issues, days when activities were affected by physical and or mental issues)

When multiple linear regression was applied, having a higher monthly income remained a significant factor which increased the scores for physical health and social health, and additional significant result for environmental health. The other factors that continued to play a role on QOL domains were: days when activities were affected by physical and or mental issues (associated with physical health and psychological health), Malay ethnicity (associated with psychological health and environmental health), being single (associated with social relationship) and days affected by mental issues (associated with social relationship).

## Discussion

This study has shown that despite having thalassaemia, the majority of the participants perceived their general health status as good. Their quality of life (QOL) scores for all domains were above 60 out of a 100 (maximum score). Our scores were almost comparable with the scores from other similar studies [24, 25]. When compared the age- and sex-matched WHOQOL-BREF scores between TDTs and non-thalassemic adults in Italy, Floris 2018 reported that thalassemia subjects had scores which were at least as good as those of non-thalassemic subjects in all domains; with TDTs obtaining the highest score (SD) at 73(19) in social relationships domain and the lowest score at 63 (14) in environmental health domain. A study by Gan 2016 reported its highest score with environmental health domain at 67.38 and lowest score with physical health domain at 62.03.

Our findings suggested that the participants most likely could have received adequate and appropriate medical treatment early in life because the majority have no other co-morbidities, have gained employment and have completed at least secondary education. Such achievements would be their main concerns and struggles in life seven to ten years ago [4, 26]. The ability to obtain higher educational level has also been used as a benchmark towards a higher social level, better QOL and a better income [25–28]. Our multiple linear regression of factors has shown that having good income may lead to an increased QOL in physical health, social health and environmental health. These predictors to a better QOL were similar to another study which reported higher odds of higher income, more financial resources, better access to healthcare and lower co-morbidities to higher education [25].

The number of unhealthy days measured by CDC HRQOL-4 in this study were lower when compared with other chronic diseases such as diabetes, respiratory problems, arthritis, heart disease and mental disorders [29, 30]. Although comparatively lower, the participants in this study needed to spend at least four days every month to care for their illness. Based on our multiple regression, any additional unhealthy days with or without limitation to activities may significantly lower the QOL scores.

The participants in this study could be considered relatively young and unmarried. Hence, this could have contributed to the comparatively “higher” QOL scores. We are uncertain whether they are able to maintain or improve their QOL further as they grow older especially when some people with TDT could have been using defective coping strategies since teenage years as reported in some studies [13, 31]. They have to learn to juggle between staying healthy with the disease and doing well with normal activities. Life-training skills, self-care management and psychological treatment have been suggested as healthcare management strategies to people with TDTs to lead an independent life with good QOL and health [32–34]. These should start during their childhood and perhaps be extended to their caregivers as indicated by other studies [35, 36]. With proper training and guidance, they may fare as well as the people without the disease [37].

Based on the small number of responses to the open question, a number of participants in this study perceived having thalassaemia as a burden to their employment and working life. Their expressions, from being discriminated against, having unsupportive colleagues and friends, being inhibited from developing to their full potentials, getting a lower salary to and being fearful of losing their employment due to their frequent time-away from work, described some restrictions they have been facing. Such challenges were similarly voiced in their thalassaemia social media platform [38]. These concerns seemed to contradict the participants' perception of a good health and QOL. Therefore, there is a need for qualitative studies such as focused group interviews to assess the validity of these concerns, their actual needs and the root cause of the issue.

Meanwhile, perhaps shared consultations with other specialized teams in the healthcare system would enable people with thalassaemia to seek support and treatment, all occurring within a same hospital visit [39–41]. This may help to reduce the demand for time off from work, and financial and transportation issues experienced by many participants. Healthy social and professional integration such as anti-stigma campaigns for mental illness [42] could be considered to create a better social and environmental support for TDTs. By recognizing this group of people as valuable assets to the community and provide them equal opportunities, they could move at a faster rate towards a full social and professional life [43]. In addition, publishing the success stories of some patients could boost not only their self-esteem but also perhaps change the degree of stigmatization on them that is still present in some communities, just like in cases of AIDS and obesity [44, 45]. As one of the participants said "I am as capable like everyone else, it's just that I need a little extra blood every month".

## Strength of the study

This study provides some insight about the well-being of the adult population with TDT in Malaysia using validated questionnaires and semi-structured questions on employment status and working life. Although there are other studies on this matter, the population of interest in these studies was more focused at selected regional places. This study captured some voices of people with TDTs with regards to the challenges they were still facing.

## Limitations of the study

The main limitation in this study is the small sample size and unequal distribution of samples collected. We faced difficulties reaching most TDTs due to various reasons such as participatory refusal, poor internet coverage and lost mails. Nevertheless, we have managed to collect some data from the two regions with the largest number of registered people with thalassaemia (31%) in Malaysia, according to the latest registry [2]. Another limitation is that we could not do any comparison with healthy adults matched by age and gender. The WHOQOL-BREF questions were also rather lengthy and sensitive, probably causing some participants to choose not to answer some questions in the sections relating to social relationship and environmental health. Perhaps another healthcare and QOL tool specially tailored for thalassaemia such as ThALI should be used [46].

## Conclusion

The majority of the participants in this study seemed to be coping with the disease, and moving into adulthood with a fairly good quality of life and comparatively lower number of unhealthy days with limitation to daily activities. Some continued to face suppression of their potential capabilities and other challenges. Their future now not only depends on how to stay healthy but also how to live a near normal life just like the general population without chronic illnesses. Future qualitative studies are needed to focus on their perceived needs and to look for more tailored supportive approaches.

## Abbreviations

QOL: Quality of life
TDT: Transfusion dependent thalassaemia
MOH: Ministry of Health
CDC: Centers for Disease Control and Prevention
WHOQOL-BREF: The international version of the instrument issued by the World Health Organization (in the English, Malay and Chinese languages)
